# Preventing Microbial Contamination during Long-Term *In Vitro* Culture of Human Granulosa-Lutein Cells: An Ultrastructural Analysis

**DOI:** 10.5402/2012/152781

**Published:** 2012-09-03

**Authors:** C. O. Campos, M. P. Bernuci, A. A. Vireque, J. R. Campos, M. F. Silva-de-Sá, M. C. Jamur, A. C. J. S. Rosa-e-Silva

**Affiliations:** ^1^Department of Obstetrics and Gynecology, Faculty of Medicine of Ribeirão Preto, University of São Paulo, 14049-900 Ribeirão Preto, SP, Brazil; ^2^Department of Cell and Molecular Biology and Pathogenic Bioagents, Faculty of Medicine of Ribeirão Preto, University of São Paulo, Ribeirão Preto, SP, Brazil

## Abstract

*Purpose.* To investigate whether the addition of antibiotic/antimycotic during human granulosa-lutein cells (GLCs) isolation and cell-plating procedures prevents microbial contamination after 144 h of culture and also evaluate the effects of contamination on GLCs ultrastructure and steroid secretion. *Methods.* GLCs obtained from five women submitted to assisted reproductive techniques (ARTs) were isolated with PBS supplemented with antibiotic/antimycotic or PBS nonsupplemented and cultured for 144 h. GLCs were evaluated by transmission electron microscopy (TEM), and estradiol (E2) and progesterone (P4) secretion was assayed by chemiluminescence. *Results.* Although no contaminating microorganisms were identified by light microscopy, TEM analyses revealed several bacterial colonies in culture dishes of GLCs isolated with only PBS. Bacterial contamination disrupted the adherence of the GLCs to the culture plate interfering with monolayer formation affecting the growth pattern of GLCs. Various cellular debris and bacteria were observed, and no organelles were found in the cytoplasm of infected cells. While bacterial contamination decreased estradiol media levels, it increased progesterone, as compared with noncontaminated group. *Conclusion.* Taken together, our data showed that the addition of a high dose of antibiotic/antimycotic during the isolation and cell-plating procedures prevents microbial contamination of long-term GLCs culture as its effects on cells growth and function *in vitro*.

## 1. Introduction

The emerging of ovarian cryopreservation and follicle/oocyte *in vitro* maturation (IVM) as a potential strategy to safeguard fertility in cancer patients [1, 2] has increased the attention of the reproductive biology researches in managing long-term ovarian cells culture. Studies using human granulosa-lutein cells (GLCs) aspirated during oocyte retrieval for *in vitro* fertilization (IVF) procedure may ultimately provide data to further development of adequate milieu for oocyte growth and maturation by coculture of these cells with immature oocytes or preantral follicles. Furthermore, physical and metabolic integrity of oocyte and surrounding granulosa cells interaction is essential for the normal course of the folliculogenesis process [3–5], which makes the managing of the *in vivo*-like granulosa cells function during culture important for the IVM approach success.

 Microbial contamination of long-term cell culture is a current concern for assisted reproductive techniques (ARTs) laboratories. Although transvaginal oocyte retrieval for IVF is considered to be relatively safe [6], occasionally microorganisms colonize culture dishes of oocytes and embryos [7, 8]. These infections are mainly caused by bacterial strains insensitive to the antibiotics used or due to yeast colonization by *Candida* species, which frequently reside in the vagina [9], or even by other normal vaginal flora, like lactobacillus [10]. As bacterial endotoxins dramatically disrupt granulosa cells growth and function *in vitro* in different species [11–13], a technique for the isolation and plating of human GLCs that prevent microbial contamination would provide a powerful tool to guarantee the long-term management of functional cells *in vitro*.

The methods in common use today to prevent microbial contamination in IVF granulosa cells culture employ the supplementation of the culture media with antibiotics and/or antimycotics [14–16]. Alternatively, the use of antibiotics and antimycotics during the initial isolation and cell-plating procedures has been also shown to reduce the potential contamination of various types of mammalian cells cultures [17]; however, the efficacy of this procedure to prevent microbial contamination of the human GLCs culture remains to be determined. 

Accordingly, the purpose of this study was to determine the feasibility of using an isolation protocol with PBS containing antibiotic/antimycotic as washing solution during the recovery of granulosa cells from the follicular aspirate during oocyte retrieval for ART in order to obtain germ-free long-term culture. After 144 hours, the cultures of human GCs were examined for morphological and functional characteristics. The use of PBS containing antibiotic/antimycotic as washing solution improved cell culture purity and retained cell functionality, which is essential for characterization of granulosa cells function *in vitro*.

## 2. Materials and Methods

### 2.1. Protocol for Ovarian Hyperstimulation

Human GLCs were obtained from five patients aged between 18 and 40 years, who presented ovulatory cycles and FSH below 12 mIU/mL. All patients were indicated for assisted reproduction procedure because of male or tubal factor infertility and agreed to sign the informed Consent. Patients were excluded if they had endometriosis or failed fertilization in previous cycles, presence of any endocrinopathies as congenital adrenal hyperplasia, hyperprolactinemia, or thyroid disorders, even if compensated. Patients using drugs known to affect reproductive or metabolic function, as treatment with glucocorticoids or antiandrogens, for at least 60 days before the beginning of the ovulation induction were also excluded. All patients were submitted to follicular aspiration for IVF procedures at the Human Reproduction Division-Department of Obstetrics and Gynecology, Faculty of Medicine of Ribeirão Preto, University of São Paulo. All procedures were approved by the Research Ethics Committee from this institution (Protocol HCRP no 7474/2008). Follicular development was achieved by treatment for 10 days with 400 mcg/day of GnRH agonist Nafarelin-Synarel, Pfizer (before ovarian hyper stimulation), followed by daily dose of 100 to 375 UI of recombinant FSH, administered subcutaneously (Gonal F, Serono Canada, Oakville, ON, Canada). Serial transvaginal ultrasonography was performed to evaluate the follicular growth. When the majority of the follicles reached 17 mm diameter or more, the patients were injected with 10,000 IU of hCG (Ovidrel, Serono, Brazil) or 250 mcg of recombinant LH (Organon). Oocyte retrieval was performed 34–36 hours after the drug administration and the follicular aspiration was guided by transvaginal ultrasound. The follicular fluid was examined by stereomicroscope, and the oocytes were identified and used for ART.

### 2.2. Cell Culture

For cell culture experiments the follicular fluid was collected in 50 mL sterile polypropylene tubes (Falcon) containing PBS or PBS supplemented with 1000 U/mL penicillin, 1000 *μ*g/mL streptomycin, and 0.25 *μ*g/mL amphotericin B (Invitrogen-Gibco/BRL, Grand Island, NY). The follicular fluid was washed with PBS in sterile polystyrene petri dish. The GLCs clusters were carefully recovered after red blood cells sedimentation. The GLCs were centrifuged at 300 ×g for 10 min at 4°C and the pellet was resuspended in TCM-199 (Invitrogen, Gibco Laboratories Life Technologies Inc., Grand Island, NY). GLCs were separated from remaining red blood cells by gradient using Histopaque-1077 (Sigma Chemical Co., St. Louis, MO, USA). After 20 min centrifugation at 500 ×g the GLCs layer was collected with Pasteur pipette from the interface between the Histopaque and the medium. The cells were then centrifuged at 500 ×g for 10 min at 4°C with 2 mL PBS, to remove the Histopaque. The cell pellet was resuspended in 100 *μ*L of culture media. Aliquots from isolated cells with or without PBS containing antibiotic/antimycotic were counted in hemocytometer. The cell viability was accessed with 0.2% trypan blue, and 1 × 10^5^ viable cells were transferred into individual wells of 24-well plates containing 200 *μ*L TCM-199 medium supplemented with 10% fetal calf serum, 100 U/mL penicillin, 100 *μ*g/mL streptomycin, and 0.025 *μ*g/mL amphotericin B (Invitrogen-Gibco/BRL). The cells were cultured at 37°C in 5% (v/v) CO_2_ in air atmosphere for 144 h, and70% of the culture media (140 *μ*L) was collected and replaced every 48 h and stored at −20°C.

### 2.3. Microbiologic Examination

Cultures were evaluated by microscopy, and further investigation for bacterial or fungal contamination was done in the microbiology laboratory according to standard procedures. Samples were inoculated on blood agar plates and (brain heart infusion) BHI for detection of bacterial, yeast, or fungal contaminations. Plates were incubated aerobically at 37°C and read within 24 h and 48 h. 

### 2.4. Electron Microscopy

After culture the GLCs were fixed in 2% glutaraldehyde, 2%formaldehyde, and 0.05%CaCl_2_ in PBS withCa^2+^/Mg^2+^.Thenthesampleswere postfixedin1%osmium tetroxide in distilled water(pH7.4),contrasted with0.5% uranyl acetate,dehydrated in ethanolPA (Merck), and embedded inEpon 812. Semithin sections (0.5–1 *μ*m thick) were stained with toluidine blue and examine by light microscopy. Ultrathin sections (60 nm) were mounted on copper grids, stained with 0.5% Uranyl acetate and lead citrate, and examine in transmission electron microscope (JEM-100CXII, JEOL).

### 2.5. Hormones Assay

Estradiol (E2) and progesterone (P4) media levels were analyzed by chemiluminescence using a commercial kit (DPC, Immulite System, Los Angeles, CA, USA), in accordance with the manufacturer's protocol. All samples were analyzed in duplicate, to minimize intra-assay variability or variability in the same assay. The intra-assays coefficients of variation were less than 10%.

### 2.6. Statistical Analysis

Data are presented as mean and SEM. Differences in E2 and P4 levels between bacteria-infected and-noninfected groups were analyzed by Student's *t* test. *P* < 0.05 was considered statistically significant.

## 3. Results

### 3.1. Morphology of Human Granulosa-Lutein Cells Is Preserved after Long-Term Culture

**Figure 1:**
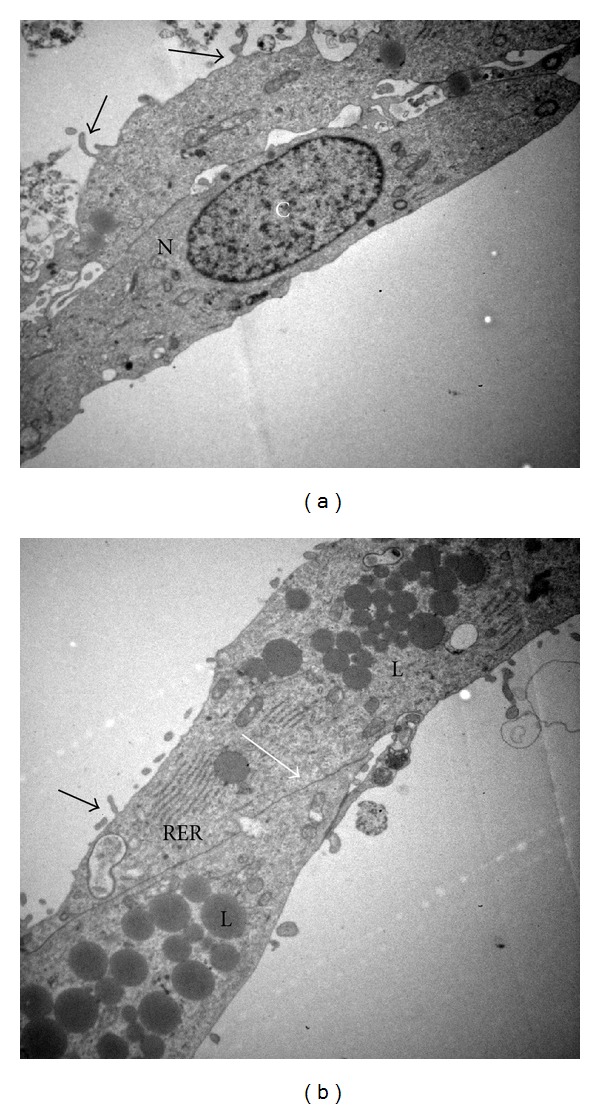
Transmission electron micrographs of human granulosa-lutein cells (GLCs) collected with PBS containing antibiotic/antimycotic cultured for 144 hs in TCM-199 medium. (a) Typical elongated health granulosa cell showing cytoplasm with organelles and nucleoplasm contained finely dispersed chromatin. (b) Typical monolayer granulosa cells culture showing contact area between two cells (white arrow). N: nuclei, C: chromatin, L: lipid droplet, RER: rough endoplasmic reticulum, black arrows: microvilli. TEM: (a) X 6,700. (b): X 8,000.

**Figure 2:**
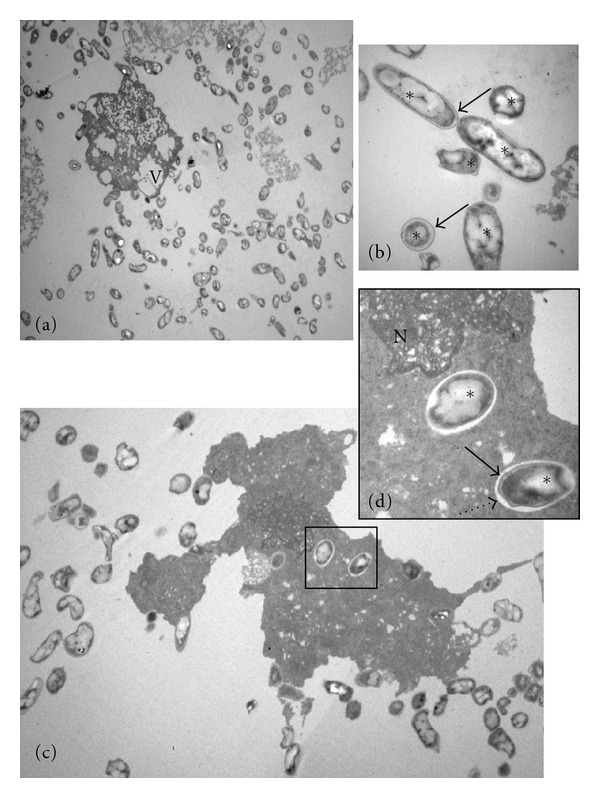
Transmission electron micrographs of human granulosa-lutein cells (GLCs) collected only with PBS cultured for 144 hs in TCM-199 medium. (a) Disrupted GLCs cytoplasm containing innumerous vacuoles and several bacteria. (b) Details of bacteria cytoplasm (*) and wall (arrows). (c) GLCs debris showing enclosed bacteria. (d) Detail of undefined and electron dense GLC nuclei, digestive vacuole (dotted arrow), and bacteria wall (arrow). N: nuclei, V: vacuoles. TEM: (a) X 27,000. (b) X 5,000. (c) X 6,700. (d) X 27,000.

Human GLCs collected with PBS containing antibiotic/antimycotic were free from bacterial contaminations after 144 h of culture. Noninfected GLCs showed their typical elongated morphology of adherent cell that grows in monolayer (Figure 1). The ultrastructural analyses showed nucleoplasm contained finely dispersed chromatin and peripheral patches of heterochromatin (Figure 1(a)) as a dense cytoplasm contained several organelles and clear contacts between cells (Figure 1(b)). Human GLCs collected only with PBS showed bacterial contaminations after 144 h in culture (Figure 2). Ultrastructural analyses showed disrupted GLCs cytoplasm containing innumerous vacuoles and bacteria (Figures 2(a) and 2(b)) as undefined nucleus (Figures 2(c) and 2(d)). Several bacteria were observed inside the cytoplasm and in the extracellular media next to the cells or the cell debris (Figure 2(c)). Before plating the cell viability was assessed by trypan blue dye exclusion, and it was 90% (data not shown).

### 3.2. Effect of Bacterial Contamination on Human Granulosa-Lutein Cells Steroid Production

**Figure 3:**
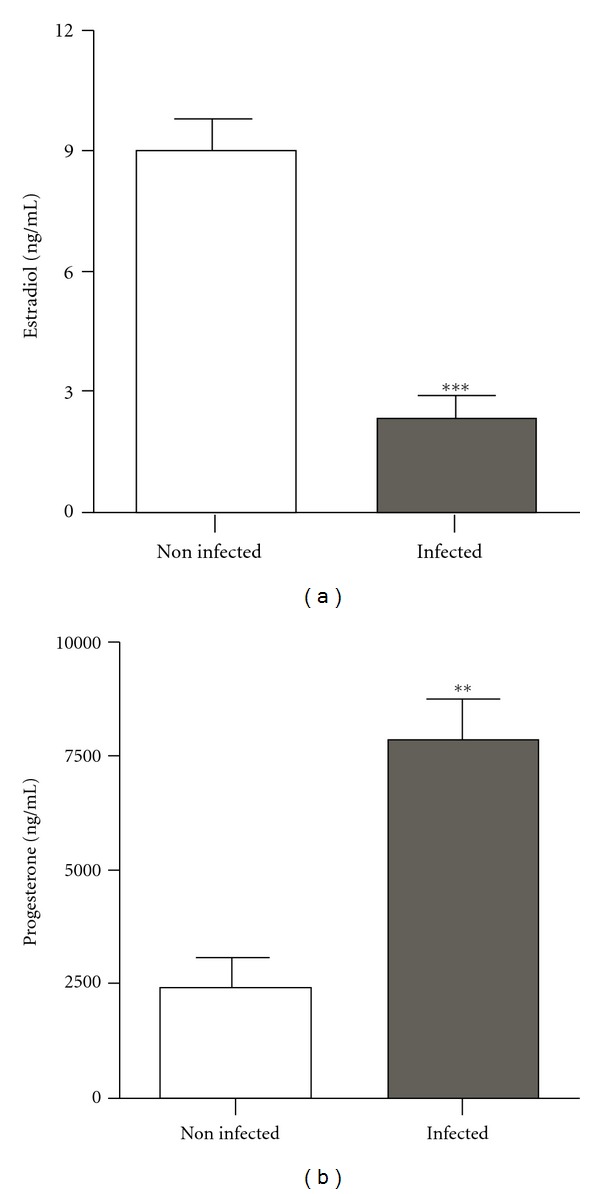
Effect of bacterial contamination on human granulosa-lutein cells (GLCs) steroid secretion. Concentrations of media estradiol (a) and progesterone (b) after 144 hours of culture. GLCs collected with PBS containing antibiotic/antimycotic (noninfected group) or collected only with PBS (infected group). Data shown as mean ± SEM; ***P < 0.0001; **P < 0.005 versus noninfected group.

Estradiol production by GLCs that were collected with PBS supplemented with antibiotic/antimycotic was higher than that produced by cells collected only with PBS (Figure 3(a)). In contrast, the progesterone production by GLCs cells collected only with PBS was higher than that produced by cells that were previously washed in PBS supplemented with antibiotic/antimycotic (Figure 3(b)).

## 4. Discussion

In this report we described an optimized protocol for isolation of human granulosa-lutein cells where the cells were rinsed in PBS containing antibiotic/antimycotic prior to the culture. Although antibiotics are routinely added to media for long-term culture of cells and tissues, to avoid bacterial contamination, it also could lead to development of resistant bacteria with slow-growing property. As the vaginal flora contains a large variety of bacterial species [10, 18] it may contaminate the sample during oocyte retrieval with transvaginal ultrasound leading to further bacteria growth into the culture system. Our electron microscopy results revealed bacterial contamination in GLCs culture that could not be seen by light microscopy. The culture GLCs with bacterial contamination showed several ultrastructural damages. 

In the present study using a mixture solution containing 10-fold of antibiotic/antimycotic in PBS to rinse the cells prior to the culture and also in the culture media was possible to culture human GLCs for 144 hours without bacterial contamination. Our results also showed that the use of antibiotic/antimycotic only in the culture media could not avoid the contamination. Indeed, infections in IVF culture dishes have been associated with bacterial strains insensitive to the antibiotics used or due to yeast colonization by Candida species, which frequently reside in the vagina [9]. As suggested by Vierck [17] the use of antibiotics and antimycotics in primary cultures of muscle and fat stem cells during the initial isolation and cell plating procedures may greatly reduce the potential for contamination of the cultures. Penicillin (100 IU/mL) and streptomycin (100 mg/mL) are the most common antibiotics used to control microorganisms in culture media, effective against a variety of gram-positive and gram-negative bacteria. Amphotericin B (0.25 mg/mL) is often added to control fungal contamination. Our finds demonstrate that it is important to rinse the cells with PBS containing antibiotic/antimycotic before placing the cells in culture. These results suggest that the contamination was acquired during the vaginal access. Moreover, these results indicated that the contamination was not acquired during the cell handily since all steps of the culture procedure such as, oocyte retrieval, cells platting, and media preparation and replacement were done with extremely care and in sterile conditions. 

We also showed that human GLCs steroidogenesis was markedly affected by bacteria contamination in the culture. These crypts contaminants may persist indefinitely in cultures causing subtle biochemical changes into the culture medium disturbing cell growth and differentiation [19]. Estradiol levels in the culture media were significantly reduced after bacterial contamination while progesterone levels increased. Similar results were shown using GLCs cultured in the presence of* Escherichia coli*, which leads to stimulation of progesterone and inhibition of estradiol production *in vitro*. Studies in different experimental models have suggested that the effects of bacterial contamination on granulosa cells function are likely mediated directly through the endotoxin, lipopolysaccharide (LPS), or indirectly through the inflammatory mediators associated with bacterial infection including cytokines such as tumor necrosis factor alpha (TNF-alpha) [11–13]. Although suppression of estradiol granulosa cells secretion by bacterial infection has been associated with downregulation of transcripts for aromatase [20], the mechanism (s) by which bacteria exert its adverse effects upon progesterone secretion remains to be determined. As granulosa cells function is pivotal during follicle development and these cells nurture the oocyte until ovulation, the maintenance of an adequate milieu for granulosa cells growth and differentiation *in vitro* is likely to be an important approach underlying ovarian follicular cells physiology. 

In conclusion, the cell isolation protocol presently used allowed the prevention of microbial contamination in long-term culture of human GLCs and the maintenance of cells growth and function *in vitro*. Thus, taken together, our results suggest that the use of antibiotics and antimycotics in wash buffer and in the culture media is essential for long-term culture of human GLCs. Future experiments will be directed to determine whether this protocol may help to develop healthy culture system for coculture of granulosa cells with immature oocytes or preantral follicles in order to obtain applicable results to fertility preservation programs and for infertility treatments.
